# Accessory tympanic plate ossicle: a new osteological entity

**DOI:** 10.1093/fsr/owae003

**Published:** 2024-01-15

**Authors:** Robert W Mann, Sittiporn Ruengdit, Karen Thompson, Kiana Miller, Scott Lozanoff

**Affiliations:** Department of Anatomy, Biochemistry, and Physiology, John A. Burns School of Medicine, The University of Hawai’i at Mānoa, Honolulu, HI, USA; Department of Forensic Medicine, Faculty of Medicine, Chiang Mai University, Chiang Mai, Thailand; Forensic Anthropology and Entomology Center, Faculty of Medicine, Chiang Mai University, Chiang Mai, Thailand; Department of Pathology, John A. Burns School of Medicine, The University of Hawai’i at Mānoa, Honolulu, HI, USA; King County Medical Examiner's Office, Seattle, WA, USA; Department of Anatomy, Biochemistry, and Physiology, John A. Burns School of Medicine, The University of Hawai’i at Mānoa, Honolulu, HI, USA

**Keywords:** forensic sciences, forensic anthropology, ossified auricular cartilage, ectopic bone, petrified ears

## Abstract

The auricular cartilage, which is typically soft and flexible, can calcify or ossify because of diseases such as diabetes mellitus, trauma, radiation therapy for cancer, and more commonly from frostbite. Calcified, ossified, or hardened auricular cartilage is a rare finding in the clinical literature and appears to be absent in the physical and forensic anthropological literature. This study examines the ossified auricular cartilage and tests whether the hypothesis can be identified in postmortem skeletonized tissue and be part of the external auditory meatus. A total of 290 crania were examined for accessory ossicles. A descriptive and interpretative analysis was performed grossly, histologically, and morphometrically to document the morphology and location of the ossicles, investigate their structure, and perform hypothesis testing. Results revealed that seven females and one male crania from a total of 290 crania (2.76%) exhibit semi-ossified auricular cartilage attached to the tympanic plate of the temporal bone. The morphology and location of the ossicles at the junction of the auricle and external auditory meatus indicate they are hardened auricular cartilage that was verified with histological observations. Regression analysis indicates that addition of the ossicle to the depth of the auditory tube significantly changes coefficient of determination (*R*^2^) with respect to cranial breadth. In conclusion, results indicate that small cartilaginous structures of the external ear may ossify forming accessory tympanic plate ossicles that potentially could be identified in skeletal remains as a new osteological entity. This report highlights the types of information that can be gained using an approach that integrates forensic anthropology, gross anatomy, and histology.

## Introduction

The temporal region of the craniofacial skeleton is a complicated composite of neural, vascular, and connective tissues that can provide significant archaeological and forensic information including the number of individuals, clinical, age estimation, and personal identification indicators [1, 2]. External ear anatomy has been extensively studied for forensic purposes. The complexity and unique morphology of the auricle has been proposed for personal identification using ear print due to its polymorphism among individuals [3]. Additionally, development of the tympanic ring has been utilized as a key indicator for estimating neonate age [1] and the presence and visibility of the oval window as a feature of ancestry [4, 5]. However, due to its complexity, identification of temporal region components can confound an examiner. In particular, the degree of connective tissue ossification can vary widely between elements, e.g. the petrous portion of the temporal bone *versus* the auditory ossicles. Additionally, the origin of the temporal bone and its four primary components including the squamous, petrous, tympanic ring, and styloid process all vary in terms of their sequence of ossification and may show independent regional variations further complicating postmortem identification [6].

The external ear, or auricle, forms the most lateral portion of the temporal complex. The auricle is of forensic interest because it can aid in victim identification [3]. The auricle arises from a set of hillocks around the first pharyngeal pouches and at the confluence of the first and second pharyngeal arches. These tissues merge to form corresponding features of the developing ear in a highly integrative fashion [7]. Thus, the external ear becomes anchored to the external auditory meatus (EAM) and provides a semi-rigid auricular cartilaginous support system for the auricle.

The outer ear consists of two components, the pinna and the oval-shaped EAM, both consisting of skin, perichondrium, and cartilage [8–10]. The auricular cartilage, a single plate of thin yellow fibrocartilage that fills the ear, is subject to a host of maladies that include hardening and inflexibility, a condition known as petrified ear [11], auricular ossificans and ectopic ossification [12], and petrified pinna [13]. Hardened auricles have been found to affect the mobile helix, antihelix, and anti-tragus [14], sometimes enabling the once flexible ear to be moved as a unit, without involving the lobule [12]. Other than in one report [15], hardening does not appear to affect the EAM.

The auricular cartilage, which is typically soft and flexible, can calcify or ossify because of disease such as diabetes mellitus, trauma (e.g. boxing or wrestling), radiation therapy for cancer [12], and more commonly from frostbite [16, 17]. Hardened auricles are more commonly found in males and individuals over 60 years of age, and bilaterally [13]. Regardless of its aetiology, antiquity, or frequency, hardened auricular cartilage is a rare finding in the clinical literature and appears to be absent in the anthropological literature. The purpose of this study is to report and thoroughly examine the rare cases of the ossified auricular cartilage, referred to in this research as accessory tympanic plate ossicles (ATPOs) and test the hypothesis that ossified auricular cartilage can be identified in postmortem skeletonized tissue, retrieved in a controlled environment, and be part of the EAM or not.

## Materials and methods

### Subjects

A total of 290 known-identity crania utilized in this study were drawn from the Mann–Labrash Osteological Collection which is part of permanent body donations with institutional approval. All activities conformed to the standard operating procedures of the Willed Body Program (WBP) at the John A. Burns School of Medicine (JABSOM), University of Hawaii, Manoa [18]. The samples belonged to 156 male and 134 female donors with mean age of 75 years.

The soft tissue was removed from the crania before being placed into separate vivara with colonies of dermestid (Dermestidae) (flesh eating) beetles to remove the remaining soft tissue. The crania were examined daily to monitor the progress of soft tissue removal. Once the skulls were free of soft tissue they were photographed and examined before being immersed in acetone to degrease them.

A descriptive and interpretative analysis was performed to document the morphology and location of each ossicle. The ATPOs were examined grossly and microscopically using a stereomicroscope (Olympus SZ40, Feasterville, PA, USA) with 12× magnification. The descriptive analysis focused on the size, shape, location, and number of ossicles metrically and morphologically. The interpretive analysis utilized a process of elimination (i.e. in medical terms, a differential diagnosis) [19] to eliminate possibilities and arrive at the most logical conclusion.

### Histology

Tissue samples were excised from the ATPOs found among 290 crania, fixed in 10% formaldehyde and decalcified (10% HCl) followed by paraffin embedding. The embedded sample was positioned, sections (6 μm) with a rotary microtome and sections were mounted on glass slides with cover slips. Routine Hematoxylin and Eosin (H&E) was used to stain the slides. Selected sections were viewed (10×, 20×, 40×) with an Olympus compound microscope (model BX 41TF, 1D11217; Olympus, Tokyo, Japan) and photographed.

### Morphometric analysis

Morphometric features of the auditory meatus derived from the crania that had an ATPO were compared with a control sample (30 crania) drawn from the Mann–Labrash Osteological Collection (WBP, JABSOM). The hypothesis tested was that the ATPO represents a detached bony portion of the tympanic plate. Alternatively, if there is a significant difference between auditory canal depth (with the ATPO included in the measurement), then the null hypothesis was that the ATPO likely represents an ossified portion of the auricle.

**Figure 1:**
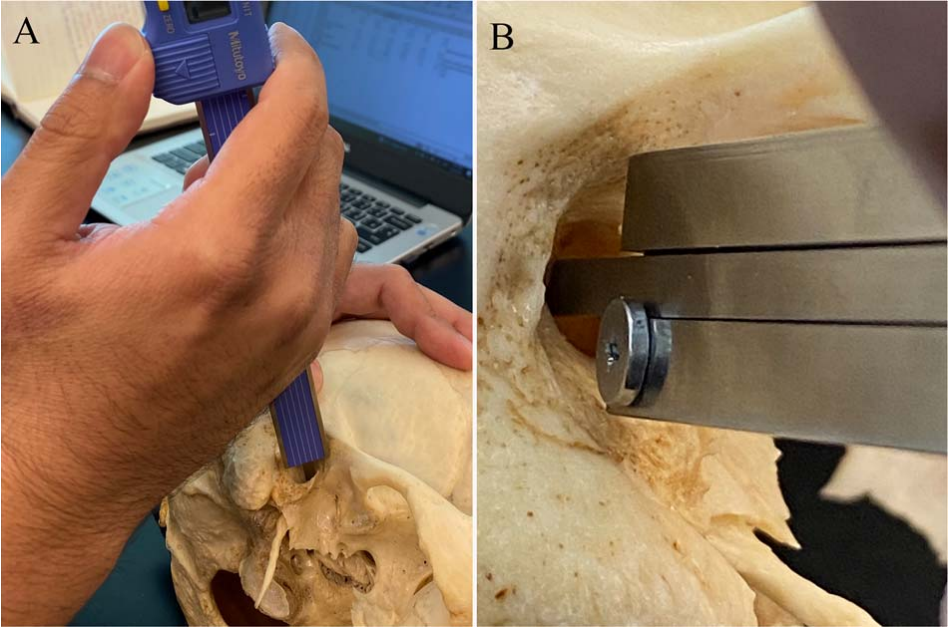
Measurement of the depth of the auditory meatus. (A) The measurement is taken by inserting the internal depth probe of the sliding caliper into the auditory meatus (B) with the caliper arms oriented along the superior and inferior portions of the oval-shaped auditory meatus.

Measurements of the ATPO thickness, obtained from only five ATPOs belonged to three individuals, were used in the regression and correlation analysis due to the deficient edge of structure in the other individuals. Depth of the auditory canal and cranial breadth were obtained from all crania that had an ATPO and the control sample using a Mitutoyo^©^ digital sliding caliper (0.1 mm) and Paleo-Tech spreading caliper (1.0 mm). Depth of the auditory canal represented the width of the tympanic plate. Specifically, it was obtained by inserting the internal depth probe of the caliper into the auditory meatus with the caliper arms oriented along the superior and inferior portions of the lateral/external portion of the oval-shaped auditory meatus until the depth probe abutted the vestibule while keeping the caliper arms in contact with the auditory process (Figure 1). Cranial breadth (euryon–euryon) was selected as a scaling factor.

Descriptive statistics were performed to obtain the mean and standard deviation of both measurements. Relationship between depth of the auditory meatus and cranial breadth, as a comparative measurement, was also tested using correlation analysis. Regression analysis was performed to determine how well the two measurements were related. A regression model was used as a statistical method to test the hypothesis mentioned earlier. If the ATPO likely represents an ossified portion of the auricle and is not part of the auditory meatus, the derived regression model would be affected when the thickness of ATPO, in medial-lateral dimension, is considered. A total of 30 individuals were randomly selected from the Mann–Labrash Osteological Collection to test interobserver error. The significance was set at *P*-value <0.05. R v.4.2.2 software [20] was used for the statistical analyses.

## Results

### General observations

A total of 13 calcified ATPOs, consisting of six left and seven right ossicles, were found in seven female and one male adult crania in a sample of 290 adult crania. Demographic data of these eight crania are tabulated in Table 1.

**Table 1:** Demographic data of the eight adult crania with calcified accessory tympanic plate ossicles (ATPOs).

**Individual**	**Sex**	**Age (years)**	**Self-reported ancestry/population affinity**	**Cause of death**	**Morphology of ATPO**			**Dimension (mm) (AP×SI×ML)**			**Weight (mg)**	
					**Right**	**Left**		**Right**	**Left**		**Right**	**Left**
1	F	83	Hawaiian/Caucasian	Metastatic breast cancer	Boomerang	Boomerang		13.2 × 6.6 × 2.1	14.3 × 10.6 × 2.4		0.071	0.085
2	F	81	Chinese/Korean	Serous carcinoma and colonic denocarcinoma	L-shape	NF		NA	NF		NA	NF
3	F	83	Filipino/Chinese/Spanish	Metastatic lung cancer	Semilunar	Semilunar		10.8 × 7.8 × 2.3	9.9 × 5.5 × 2.3		0.047	0.032
4	F	74	Chinese/Korean	Kidney disease	L-shape	L-shape		12.5 × 6.2 × 3.1	11.1 × 6.0 × 3.0		0.041	0.051
5	F	32	European	Complications associated with Rett disease	Rectangular	NF		6.2 × 12.9 × 2.3	NF		NA	NF
6	M	66	African-American	Cancer (sarcoma)	NF	Elongated		NF	8.4 × 3.1 × 3.9		NF	NA
7	F	102	Japanese	Pneumonia (Alzheimer’s disease)	Semi-lunar and linear	Semi-lunar and linear		Semilunar: 13.0 × 13.0 × 2.0 Linear: 18.0	Semilunar: 11.0 × 11.0 × 2.0 Linear: 18.0		NA	NA
8	F	80	Korean	Colon cancer	L-shape	S-shape		17.2 × 10.2 × 2.1	16.6 × 5.0 × 3.4		NA	NA

AP: anterior–posterior; SI: superior–inferior; ML: medial–lateral; NF: ATPO was not found; NA: measurement was not applied for that ATPO because it was still attached to the external auditory meatus.

Five individuals exhibited bilateral ATPOs and three individuals exhibited unilateral ATPOs. An individual which had bilateral cartilaginous semi-lunar structures with no histological evidence of calcification, suggesting that they are normal cartilage inferior to the auricle, was also investigated for a comparative sample. All the ATPOs were held to the lateral surface of the tympanic plate by a thin bridge of translucent connective tissue. Most ATPOs were either lighter or darker than the bone around them and all were translucent when transilluminated (backlit) with a small Logan desk top light box.

### Osteological and histological features

#### Individual 1 (bilateral ossicles; left ossicle)

Individual 1 exhibited ossicles attached to the lateral margin of the left tympanic plate of the temporal bones at the junction of the auricle and EAM. The cranium of Individual 1 was examined on the third day in the beetle colony. By Day 4, the beetles had disarticulated and dislodged this accessory bone, which was found in the frass (beetle excrement), several centimetres from the temporal bone. No accessory bone was seen attached to the right temporal bone.

**Figure 2:**
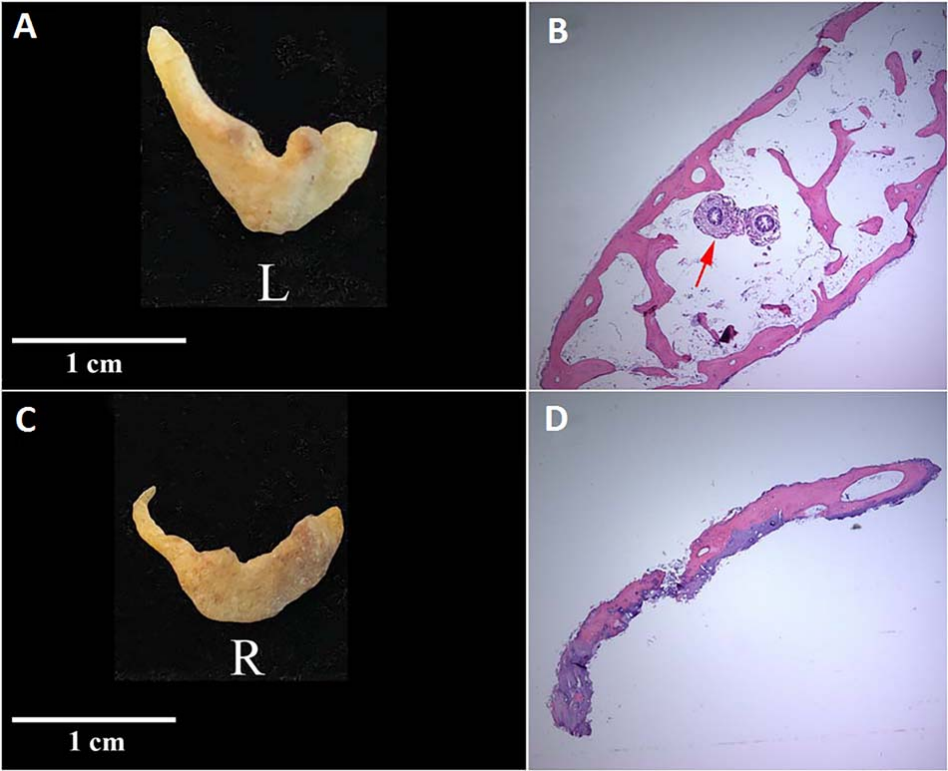
External view of left (A) and right (C) calcified auricles from Individual 1. Histology of the left (B) and right (D) ossicles reveals ossification with empty lacunae (B; arrow) and an insect, probably a dermestid beetle, inside the marrow cavity of the left ossicle.

**Figure 3:**
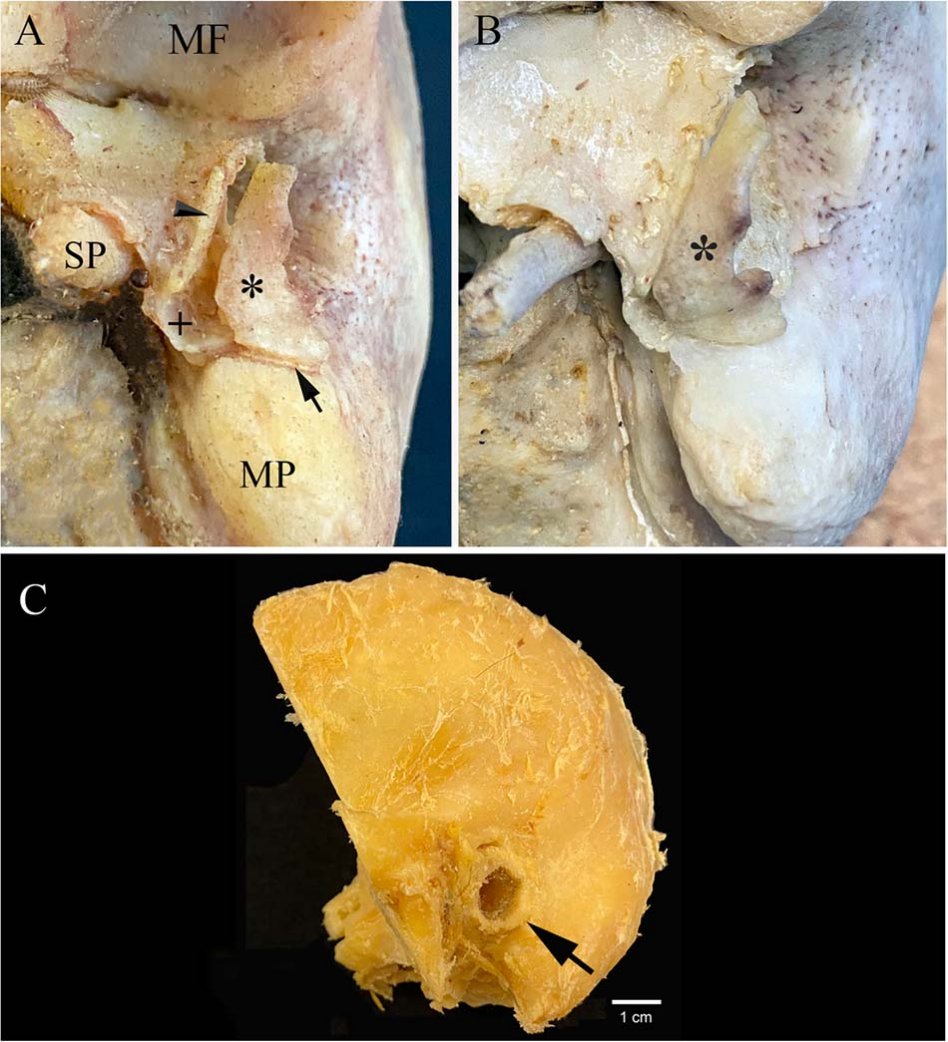
(A) *In situ* inferior view of the left ossicle (*) on Day 3 in the dermestid beetle colony from Individual 1. Note the site of attachment (arrowhead) of this ossicle along the anterior–inferior margin of the tympanic plate (+) and its attachment to the anterior surface of the mastoid process by a thin bridge of connective tissue (arrow) along the tympanomastoid fissure (MF: mandibular fossa; SP: styloid process; MP: mastoid process). (B) Inferior view of the reattached (with clear wax) ossicle (*) exactly following the contour of the tympanic plate. (C) Left temporal bone showing attachment of the auricle (arrow) in an adult female that is similar in size and shape to some of the accessory tympanic plate ossicles (ATPOs) in these eight individuals.

The left ossicle (Figures 2A and B and 3A and B) was first noticed while attached to the mastoid process by soft tissue. This bone, which was pulled away from the tympanic plate postmortem (Figure 2A), mirrors the size, shape, and contour of the irregular surface along the anterior margin of the left tympanic plate. The posterior part of the left tympanic plate fuses to the mastoid process with the tympanic ossicle lying lateral to it and attached along its inferior margin to the anterior surface of the mastoid process by a thin bridge of connective tissue (Figure 3A). The ossicle is translucent and the same consistency and density throughout. It is convex laterally and concave medially, exhibits relatively flat anterior and posterior ends that at first look broken, but are not, and is firm and inflexible. The ossicle extends along the length of the anterior–inferior margin of the EAM at the junction of the auricle mirroring the size, shape, and location in a dissected temporal bone (Figure 3C). A sample of this ossicle was subjected to histological examination (Figure 2B) revealing that it is comprised entirely of mature trabecular bone with a marrow cavity. No other ossified portions of the left ear were found with the cranium.

#### Individual 1 (right ossicle)

Five months after the left accessory ossicle was found, a disarticulated ossicle similar in morphology to the one attached to the left temporal plate was found deep in the beetle frass, about 25 cm from the right temporal bone, likely having been moved by the beetles while feeding. This loose bone was found to precisely articulate with the right tympanic plate of the temporal bone.

**Figure 4:**
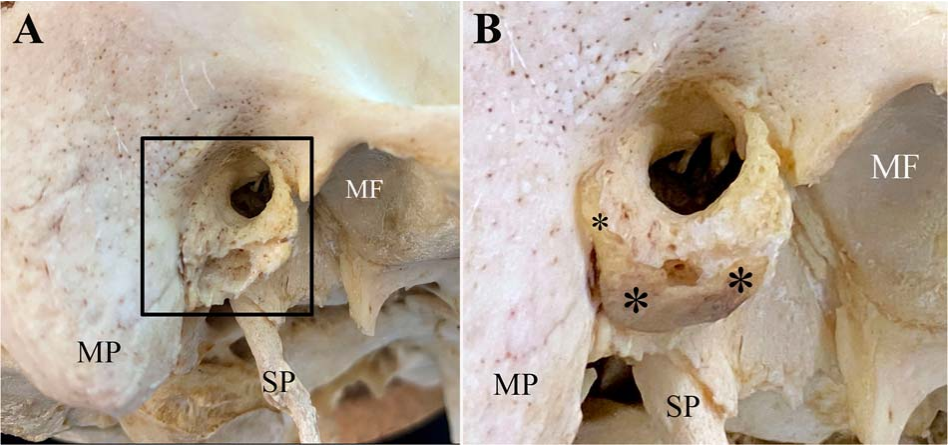
Right temporal bone without (A) and (B) with the reattached portion of ossified right auricular cartilage in Individual 1. The auditory process consists of an incomplete bony ring with a roughened surface that is open along its superior portion forming the anterior wall, floor, and part of the posterior wall of the external auditory meatus. (A) Note the rough lateral surface of the auditory process (square) without the ossified cartilage (MP: mastoid process; SP: styloid process; MF: mandibular fossa). (B) The reattached (with clear wax) ossified cartilage (*) precisely follows the contour of the auditory process allowing accurate rearticulation.

The right ossicle (Figure 2C and D and Figure 4) is fragile and translucent, is the same consistency throughout, and exhibits no evidence of osseous fusion with the tympanic plate. The anterior tip of this ossicle is wider and flatter than its posterior end and both ends curve medially with the posterior tip tapering to a point. Histology (Figure 2D) reveals that the ossicle is partially cartilaginous and partially ossified. No marrow cavity was present on the right. No additional segments of the ossicle were found with the skull.

#### Individual 2 (unilateral right ossicle)

**Figure 5:**
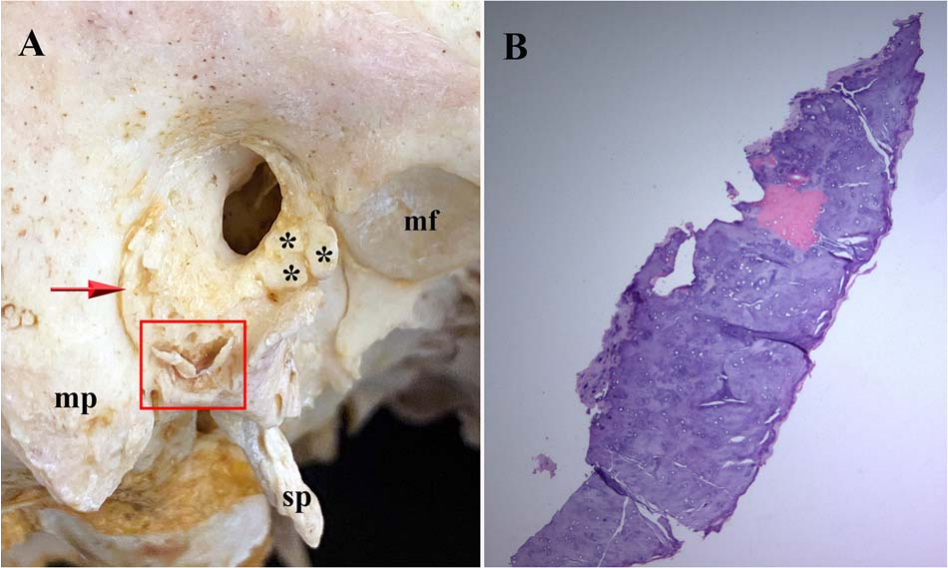
(A) Right temporal bone of an 81-year-old female (Individual 2) with a L-shaped calcification (square) along the inferior margin of the tympanic plate (mf: mandibular fossa; sp: styloid process; mp: mastoid process; *: bony growths). This ossified feature is attached to the cortex with connective tissue. The arrow indicates a semicircular ridge of bone that is part of the tympanic plate and is not calcified cartilage. (B) Histological examination of the anterior portion of the “L” reveals that this L-shaped feature consists predominantly of cartilage with some mineralization and focal ossification.

Individual 2 exhibits an accessory ossicle (Figure 5A) along the inferior portion of the right tympanic plate at the junction of the auricle and the EAM. This feature was observed after the cranium was removed from the beetle colony. It is formed by two ridges that connect to one another inferiorly and to the temporal bone by connective tissue, with no osseous fusion, along a shallow trough of the tympanic plate. There is a semicircular ridge of bone (Figure 5A) along the posterior margin of the tympanic plate that initially appeared to be calcified cartilage. That ridge is the margin of the tympanic plate. The lateral portion of the tympanic plate exhibits a roughened surface and several bony growths (Figure 5A) are firmly attached and integrated with its anterior margin. Histology (Figure 5B) revealed that the features are comprised mostly of cartilage with some mineralization and focal ossification.

#### Individual 3 (bilateral ossicles; right ossicle)

**Figure 6:**
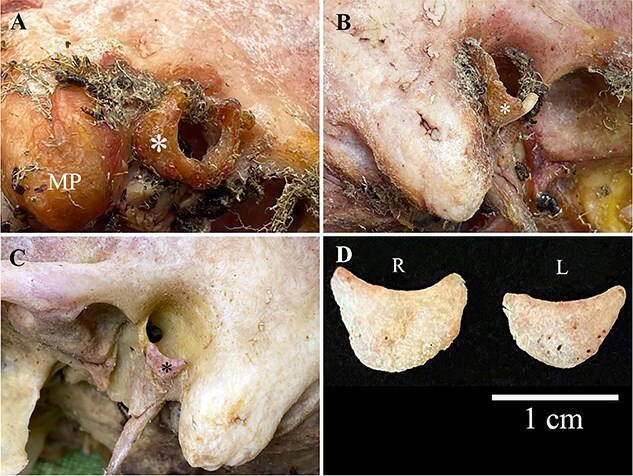
(A) Lateral view of auricular soft tissue (*) attached to the right temporal bone (MP: mastoid process) in Individual 3 after 24 h in the beetle colony, (B) partially exposed right accessory tympanic plate ossicle (ATPO) (*) *in situ* after 48 h in the beetle colony, (C) rearticulated left ATPO (*) after finding it in the beetle colony 8 days later and (D) comparison of the features of the right (R) and left (L) ATPOs.

Examination of this individual’s skull *in situ* after 24 h in the beetle colony revealed a U-shaped area of soft tissue (Figure 6A) encasing an ossified cartilage (Figure 6B). Examination of the skull after 48 h in the beetle colony revealed a piece of hardened (ossified) cartilage loosely attached to the lateral border of the right tympanic plate (Figure 6C). This ossified cartilage is convex externally and concave internally (Figure 6D) where it attaches to the lateral surface of the tympanic plate. The ossicle is translucent and its surface is slightly rough to the touch.

#### Individual 3 (left ossicle)

An ossified cartilage (Figure 6D) was found in the frass (beetle excrement; beetles are known to dislodge and move small bones out of anatomical position during feeding) 8 days after noting the right ossicle while cleaning the beetle tank. This ossicle was found to articulate with the external/lateral surface of the left tympanic plate.

#### Individual 4 (bilateral ossicles; right ossicle)

**Figure 7:**
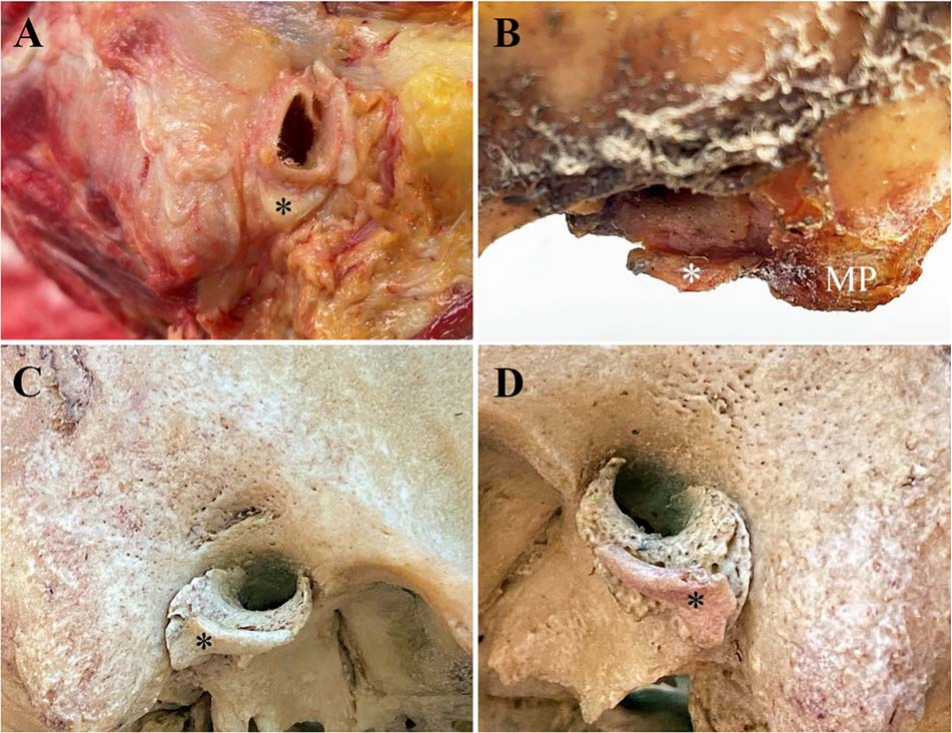
(A) Right temporal bone (Individual 4) showing a semilunar-shaped area of light-coloured soft tissue (*) encompassing (C) an L-shaped rearticulated accessory tympanic plate ossicle (ATPO) (*). (B) Left temporal bone *in situ* with an L-shaped and (D) rearticulated ATPO (*) loosely attached and following the contour of the inferior margin of the tympanic plate (MP: mastoid process).

A light–coloured area of soft tissue (Figure 7A) encasing a hard mass was noted along the inferior margin of the right auricle during removal of the soft tissue. Over a period of a few days the dermestid beetles exposed an ossicle (Figure 7C) attached to the lateral margin of the right tympanic plate by a thin layer of connective tissue. The ossicle has a rounded convex lateral surface and a concave medial surface that follows the contour of the inferior–lateral surface of the tympanic plate. The ossicle has a pointed anterior end and a wide, blunt posterior end that ended along the roughened posterior margin of the tympanic plate.

#### Individual 4 (left ossicle)

The left ossicle was noted *in situ* while removing the soft tissue of the ear (Figure 7B). A hard mass encased in light–coloured soft tissue was noted along the inferior margin of the left auricle prior to putting the cranium in the beetle colony. This ossicle (Figure 7D) was loosely attached along the inferior–lateral margin of the tympanic plate by a thin layer of connective tissue that when palpated could be moved a few millimetres in any direction. The left ossicle has a convex lateral surface and a concave medial surface where it attaches to the tympanic plate. The ossicle has a pointed anterior end and a wide, flat posterior end that attaches along the roughened posterior margin of the tympanic plate.

**Figure 8:**
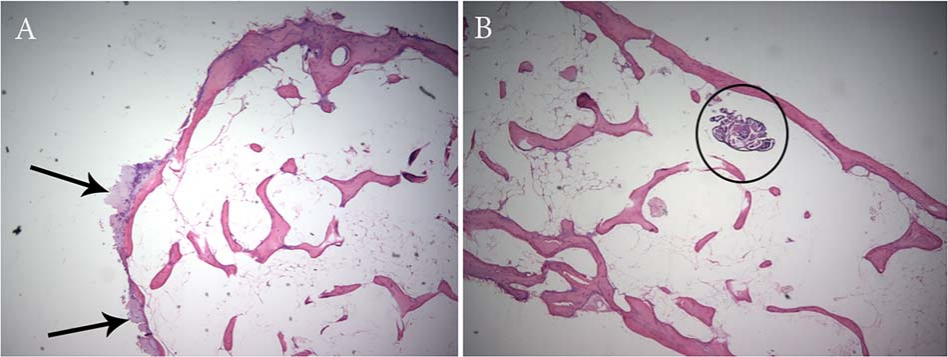
(A) Histology of the left accessory tympanic plate ossicles (Individual 4) revealed peripheral ossification (arrows) with bony trabeculae. (B) A small insect (circle) was found trapped in the marrow space.

Histological examination of the left semilunar ATPO revealed peripheral ossification with bony trabeculae and a fatty marrow cavity internally (Figure 8). There is a focus of cartilage on the surface of the ossicle (Figure 8A) and a small insect was found trapped in the marrow space (Figure 8B).

#### Individual 5 (unilateral ossicle; right ossicle)

**Figure 9:**
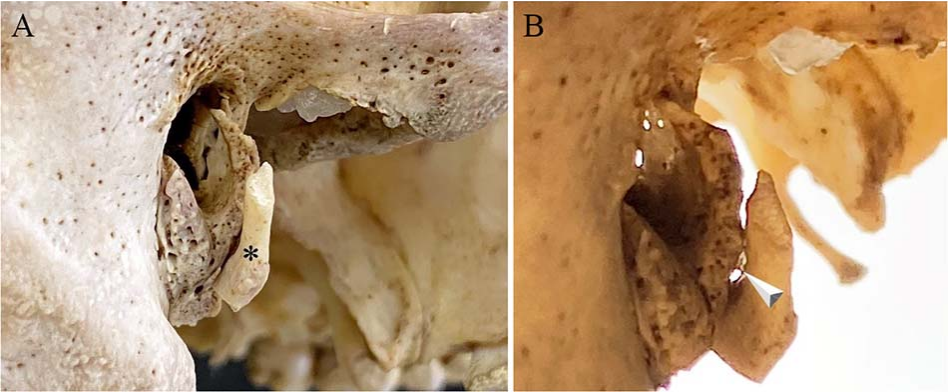
(A) Ossified cartilage (*) attached *in situ* to the anterior–inferior margin of the right tympanic plate (Individual 5). Note that the ossicle follows the contour of the tympanic plate, is lighter in colour and smoother than the adjacent bone and (B) is attached to the tympanic plate by a thin, translucent bridge of connective tissue (arrowhead).

An ossified cartilage is loosely attached to the lateral margin of the right tympanic plate (Figure 9). The ossicle is translucent when backlit (Figure 9B) and lighter in colour than the surrounding bone. This ossicle follows the contour of the tympanic plate (Figure 9B) and is attached to the anterior–inferior margin of the tympanic plate by a thin bridge of translucent connective tissue. No ossicle was found adhering to the left tympanic plate and no loose ossicle was found in the beetle colony, suggesting that this ossicle was present unilateral.

#### Individual 6 (unilateral ossicle; left ossicle)

**Figure 10:**
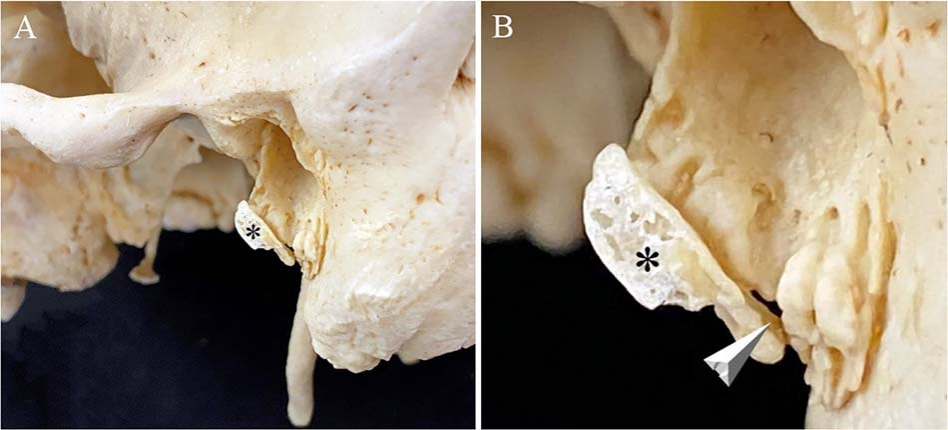
(A) Accessory ossicle (*) attached *in situ* along the anterior–inferior margin of the left tympanic plate in the male and (B) magnified view of the ossicle (*) showing a thin bridge of translucent connective tissue (arrowhead) attaching it to the tympanic plate (Individual 6).

An ossicle is attached to the anterior–inferior surface of the left tympanic plate (Figure 10). The ossicle is firmly attached by a thin (~1 mm) layer of dried/hardened connective tissue that is visible under magnification and backlighting. No evidence of an ossicle was noted along the right tympanic plate and no loose ossicle was found in the beetle colony.

#### Individual 7 (bilateral ossicles)

**Figure 11:**
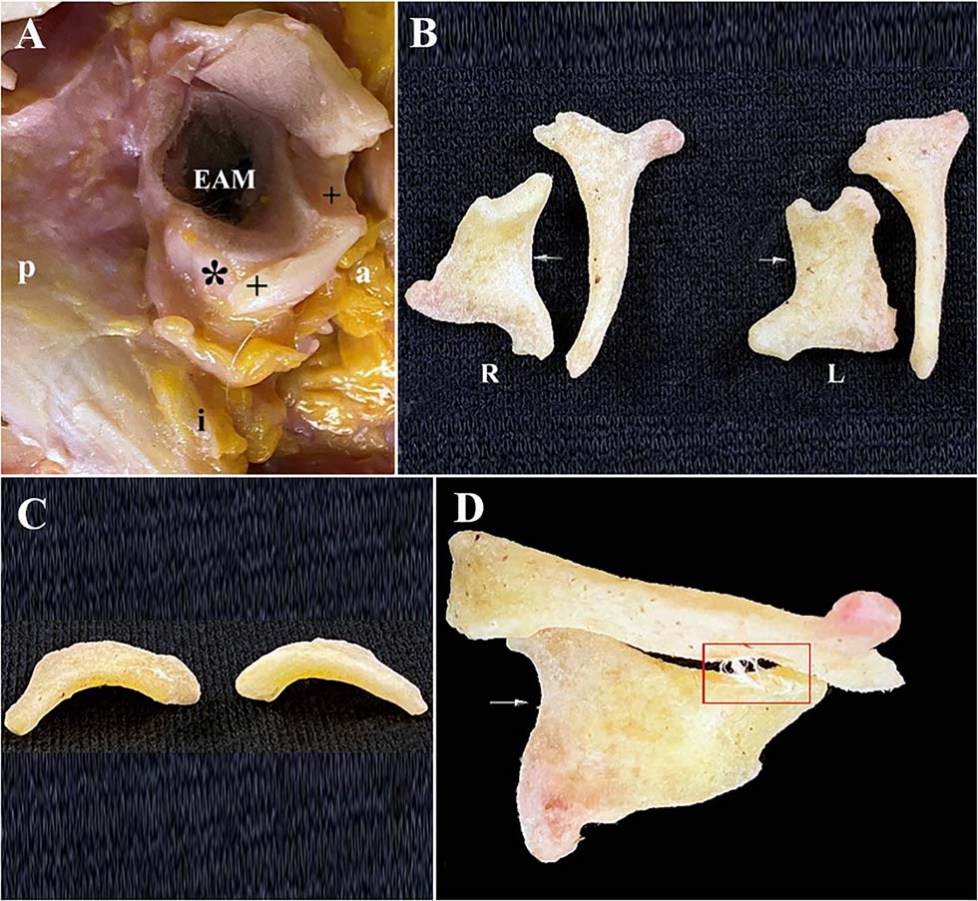
(A) Right ear showing cut (scalpel) soft cartilage (+) adjacent to a semilunar accessory tympanic plate ossicles (ATPO) (*) *in situ* and its position in relation to the external auditory meatus. (B) Symmetrical right and left calcified semilunar ATPOs and linear ATPOs in a 102-year-old Japanese female (Individual 7). The curved edge (arrows) of the two semilunar ATPOs is shown in “C”. (C) Superior view of the semilunar ATPOs showing their symmetry, curvature, and thickness (~2 mm) that joined the lateral surface of the tympanic plate forming part of the oval-shaped external auditory meatus. (D) Left semilunar and linear ATPOs joined by a thin bridge of connective tissue (rectangle) that the dermestid beetles had yet to consume and the blunt and curved edge (arrow) forming the lateral/external-most part of the semilunar ATPO.

Individual 7 is a 102-year-old Japanese female with bilateral calcified ATPOs attached to both EAM. A slightly mobile object was noted during removal of the ears (Figure 11A) and later found to be a shield-like ATPO and a T-shaped ATPO encased in soft tissue and attached along the lateral-most part of the EAM (Figure 11B–D). There was one shield and one linear ATPO (Figure 11B) attached to one another by a thin bridge of connective tissue (Figure 11D) that dermestid beetles had yet consumed. When viewed on end, the shield-like ATPOs had an arched/semilunar component consisting of a concave surface facing the EAM and a convex surface external to the auditory meatus (Figure 11C).

#### Individual 8 (bilateral ossicles)

**Figure 12:**
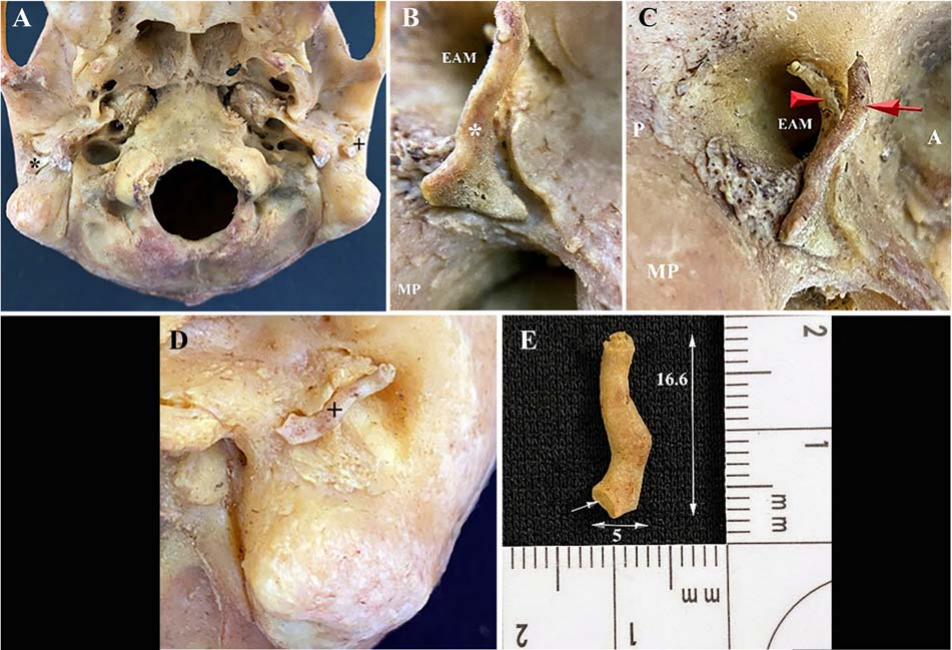
(A–B) Individual 8 showing bilateral accessory tympanic plate ossicles (ATPOs) consisting of an L-shaped right ATPO (*) and (D–E) an S-shaped left ATPO (+) (rearticulated for photography) along the anterior–inferior margin of the external auditory meatus. (A–C) The right ATPO is firmly attached to a trough in the tympanic plate by a thin bridge of connective tissue. (B–C) Note that the right ATPO (*) appears to have rotated and moved slightly out of anatomical position, possibly due to the loss of soft tissue and subsequent drying. (D–E) Note the oval facet (single arrow) at the base of this ATPO that articulated with the temporal bone and not another segment of the ATPO. EAM: external auditory meatus; MP: mastoid process; A: anterior, P: posterior; S: superior; red arrow: ATPO; red arrowhead: anterior margin of the EAM.

This individual exhibits bilateral ATPOs (Figure 12). The right ATPO is firmly attached to the tympanic plate along a shallow trough by a thin bridge of connective tissue that the dermestid beetles could not access to separate it from the temporal bone (Figure 12A–C). Although the right ATPO is held firmly in place and immobile, it is possible that it may have rotated slightly out of position (Figure 12C), extending anterior to the EAM, due to the loss of overlying soft tissues and subsequent drying while in the beetle colony. The left ATPO (Figure 12A, D, and E) could be mistaken for a temporal styloid process, both of which are unbroken and attached in this individual. The articulated right ATPO became visible after 11 days in the dermestid beetle colony and the disarticulate left ATPO was found in the frass after 11 days in the beetle colony.

### Comparative normal anatomy of the auricle and temporal bone

**Figure 13:**
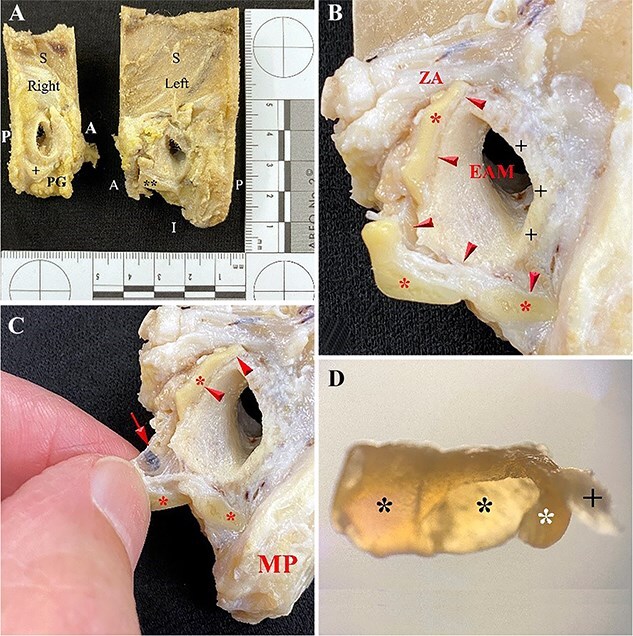
Normal anatomy of the left ear in a 72-year-old European (White) male with L-shaped cartilage (B) along the anterior and inferior rim of the external auditory meatus (EAM). (A–B) This cartilage is separated along its length from the EAM by a thin bridge of connective tissue (arrowheads) (A: anterior; P: posterior; S: superior; I: inferior; PG: parotid gland; +: semilunar cartilage; **: L-shaped cartilage). The vertical part of the L extends to the concave inferior surface of the zygomatic arch (ZA) and inferiorly and posteriorly to the anterior surface of the mastoid process (MP). (C–D) The angled portion of this cartilage grossly revealed fibrocartilage (*) and a thin, translucent layer of connective tissue (+) (20×).

This comparative sample shows the normal anatomy of the auricle and temporal bone in a 72-year-old European (White) male with uncalcified cartilage in both auricles (Figure 13). The left ear exhibits an L-shaped area of cartilage along the anterior and inferior margin of the EAM (Figure 13B–D). The cartilage in the left ear is smooth, hard and with force can be cut with a scalpel. This L-shaped cartilage moves as one unit when palpated and extends anteriorly from below the zygomatic arch to the anterior surface of the mastoid process (Figure 13B). A narrow bridge of connective tissue (Figure 13B and C) joins this cartilage to the softer whiter, and more flexible cartilage lining the EAM. The L-shaped cartilage consists of two segments (Figure 13C) joined end-to-end by a thin bridge of connective tissue along the horizontal segment of the L. The angled portion of this cartilage was air dried for 3 days and transilluminated with a microscope at 20× that revealed dense yellow cartilage (Figure 13D) and a thin area of translucent connective tissue that connected the cartilage to the EAM that is visible in Figure 13B and C. There is no evidence of hardened cartilage along the posterior margin of the EAM. The right ear exhibits a semilunar feature consisting of cartilage that measures 11 mm × 6 mm (anterior–posterior and superior–inferior, respectively) attached to the inferior margin of the EAM (Figure 13A).

### Morphometric analysis

Descriptive statistics of auditory canal depth (bilateral) and cranial breadth obtained from 38 crania are tabulated in Table 2. Results of a paired sample *t*-test demonstrates that the auditory canal depth has no statistically significant difference between left and right sides (*P* >0.05). There is also no statistically significant difference (*P* >0.05) in these measurements obtained from two observers (paired *t*-test for interobserver error).

**Table 2:** Descriptive statistics of the auditory canal depth and cranial breadth of 38 crania^*^.

**Measurement**	**Mean±SD** ** (mm)**	**Min** ** (mm)**	**Max** ** (mm)**
Auditory canal depth (right)	17.9±0.2	14.2	22.3
Auditory canal depth (left)	18.0±0.2	13.1	21.9
Cranial breadth	138.9±6.5	125.0	153.0

SD: standard deviation; Min: minimum; Max: maximum.

^*^Measurements of eight accessory tympanic plate ossicle individuals were included in the sample.

**Figure 14:**
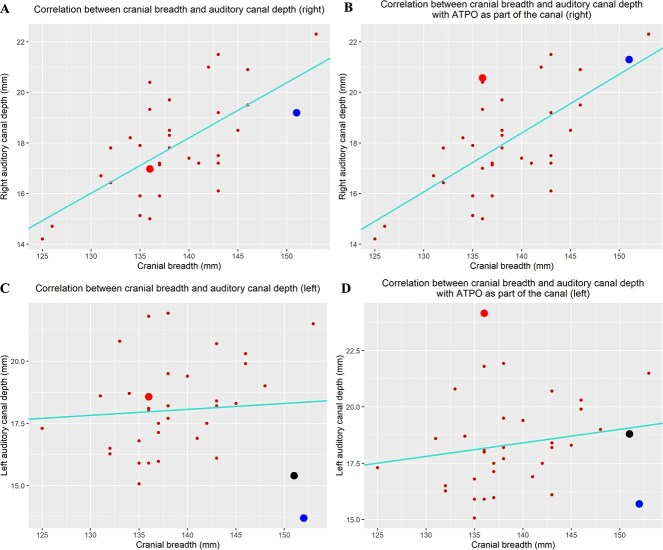
Correlation plots between cranial breadth and auditory canal depth. The big red (bottom and top for A and B, respectively; top for C and D), black (middle for C and D), and blue dots (top for A and B and bottom for C and D) represent coordinates measurements of Individual 1, 7, and 8, respectively. ATPO: accessory tympanic plate ossicle.

To test the hypothesis that the ATPO is not likely a component of the EAM, Figure 14 graphically and statistically illustrates the difference between two scenarios: (1) that ATPO was not considered to be part of the auditory meatus (Figure 14A and C) and (2) that the ATPO was part of the auditory meatus (Figure 14B and D). Cranial breadth was utilized as a comparative measurement. As shown in Figure 14, the correlation between cranial breadth and auditory canal depth was plotted for each cranium. A regression model was performed to determine the reliability of cranial breadth (*x*-axis) to predict auditory canal depth (*y*-axis). Coefficient of determination (*R*^2^) was statistical significantly altered when ATPOs were included as part of the auditory canal depth for both sides. There was also a distinct difference between the coordinate of all individuals in the graphs in both scenarios.

## Discussion

Development of the temporal bone results in osseous fusion of the tympanic ring to the squamous, then to the inferior tympanic cavity, with the remainder of the ring fusing to the petrous portion by about age one year postnatal [6]. Growth of the petrous and tympanic ring dramatically changes the orientation of the EAM in the foetus from nearly horizontal and inferior with respect to the temporal region, to vertical and lateral to the temporal bone by 4 to 5 years postnatal [21, 22]. DiBartolomeo [12] provides a radiographic example of bilateral ossification occurring in the cartilage at the junction of the auricle and EAM that is similar to the eight individuals described in this report. The tympanic ring forms as an intramembranous bone without a circumferential suture [23]. Given this developmental sequence, separation of the lateral portion of the auditory bone, with or without the tympanic ring, is unlikely.

The postmortem recovery of small elements of the skeleton and calcified laryngeal cartilage such as the hyoid bone, cricoid, and calcified tracheal rings can contribute information in establishing the age, sex, perimortem trauma, and minimum number of individuals in an archaeological or forensic anthropological context [24]. Hardened auricular cartilage, if recovered with human remains, could provide additional information on the health, disease, and longevity of individuals and groups in ancient and contemporary populations.

The presence of multiple ATPOs in a sample of 290 contemporary crania in a controlled laboratory environment suggests that similar ossicles exist in archaeological and forensic contexts. An awareness that this ossicle exists and a familiarity with its possible size and shape (Table 1) as well as texture and properties of calcified or ossified bones can increase the potential of encountering them in a standard recovery process. Knowing that these ossicles may be present in decomposed or skeletonized human remains serves as a reminder to utilize careful sifting and recovery protocols as an ATPO can resemble foetal skeletal remains, ear ossicles, sesamoids, and small fragmented bones. Additionally, examination of the lateral surface of the tympanic plate may provide evidence that an ATPO was present and dislodged postmortem. The presence of an enlarged or extended margin and roughened surface along the lateral margin of the EAM, especially on the anterior–inferior position (Figure 4), might indicate the presence of an ATPO. The possible recovery of ATPOs in archaeologically or forensically derived human remains can further our knowledge of the geographical and temporal history and frequency and possible correlation with sex, age, and ancestry of this previously unreported entity and may provide information leading to personal identification.

The virtual absence of reports of hardened auricular cartilage in the anthropological and archaeological literature could be the result of acidic soil (pH 7 or below), necrophagous insects such as dermestid beetles, or possibly by being overlooked, unrecognized, or misidentified during excavation or laboratory examination. The authors have witnessed dermestid beetles in the laboratory consuming hardened costal cartilage that results in the loss of some calcified or ossified skeletal elements. These and other variables, therefore, could result in the underreporting of ossified elements in human remains, especially those that have been buried in soil.

Two individuals in this study with ATPOs are worthy of comment because they had known genetic and developmental conditions that possibly could have influenced the development of ossified auricular cartilage. The youngest individual in this study with an ATPO is a 32-year-old female (Individual 5) who died as a result of Rett syndrome, a rare genetic disorder that affects about 1 in 10 000 female live births [25]. This female exhibited >30 skeletal anomalies including multiple bipartitions of bone in the hands and feet, right-sided positional plagiocephaly from lying on her right side, severe scoliosis, and several other rare and even possibly unique osseous features. The male (Individual 6) in this study with an ATPO exhibited an unusually large and broad hard palate and misaligned and horizontally oriented teeth because of tongue thrusting (Personal Communication Dr. J DeMeo 2022) involving repetitive movements of the head and neck. This elderly male also exhibited extremely thick and long temporal styloid processes, the right measuring 93 mm in length, a condition due to calcification or ossification of the styloid chain due in part to variations in embryological development [26, 27]. These two individuals were included in this study because they have ATPOs, without consideration of any underlying genetic or developmental condition that may or may not have influenced the development of ATPOs.

The authors used a process of elimination (differential diagnosis) based on gross examination, histology, embryology, and hypothesis testing to identify and explain the origin of the 13 ATPOs in these seven females and one male. Possible explanations for their development included accessory centres of ossification, unfused (intramembranous) tympanic rings, and hardened auricular cartilage. The possibility that the ossicles were accessory centres of ossification was eliminated since there are no known additional centres of ossification situated between the tympanic ring and the auricles. The possibility that the ossicles were unfused tympanic rings is not considered because the embryonic development of the temporal bone can only be achieved if the tympanic ring is attached to the petrous part of the temporal bone, which it was in these eight individuals. The ossicles were hardened auricular fibrocartilage, more consistent in texture, appearance, coloration, and density to ossified thyroid or cricoid cartilage than a sesamoid bone or other accessory ossicle, as a result of calcification or ossification. This was the only remaining reasonable possibility to explain the formation of ectopic bone along the perimeter of the EAM. The comparative sample in this study, e.g. exhibited bilateral semilunar features that histologically were shown to be fibrous tissue without any evidence of calcification, suggesting that most or all of the ATPOs in this paper were segments of fibrocartilage that calcified. Several individuals in this sample exhibited accessory ossicles that differed in colour, being lighter or darker (regardless of whether immersed in acetone) than the surrounding bone, which may be a result of their specific composition or density compared with the rest of the temporal bone. All the findings in this study suggest that what the authors refer to as *ATPOs* in these eight individuals are hardened auricular fibrocartilage of uncertain and as of yet unknown aetiology. Furthermore, these ATPOs are possibly sex-related entities since they belonged to seven out of 134 females (5.22%), while another one ATPO skull belonged to one from a total of 156 males (0.64%), although they are not statistically significant different (*P* >0.05) among ATPO cases found in male and female samples based on Chi-squared test. Among the eight ATPO cases, all except the 32-year-old with Rett’s syndrome, were over 60 years of age. This might reflect the age distribution of the donated skeletal samples in the Mann–Labrash Osteological Collection which demonstrates a mean age of 75.4 ± 15.0 years. Therefore, it cannot yet be concluded that ATPOs are typically present in the elderly. Although the majority of the donated skeletal remains in the Mann–Labrash Osteological Collection are European and individuals in the collection without calcified ATPOs (Table 3), only one European female was found with an ATPO. In comparison, five out of the eight ATPO cases were Asian (Table 1). Further research into the ATPO may reveal it to be a predominately Asian trait or feature that could possibly be used for ancestry or population affinity estimation.

**Table 3:** Demographic data of adult crania without calcified accessory tympanic plate ossicles.

**Ancestry/population affinity**	**Male (*n*, %)**	**Female (*n*, %)**	**Total (*n*, %)**
African	1 (0.35)	0 (0)	1 (0.35)
African-American	7 (2.48)	5 (1.77)	12 (4.26)
Asian	37 (13.12)	33 (11.70)	70 (24.82)
European	96 (34.04)	77 (27.30)	173 (61.35)
Latin-American	2 (0.71)	1 (0.35)	3 (1.06)
Native American	1 (0.35)	0 (0)	1 (0.35)
Pacific Islander	12 (4.26)	10 (3.55)	22 (7.80)
Total	156 (55.32)	126 (44.68)	282 (100)

Numbers are rounded so the percentages may not add up to 100%.

The authors named this newly reported structure “ATPO” based on a combination of variables including its location, anatomy, and histological features. For example, it is an accessory ossicle/bone because it forms lateral to the tympanic plate and, other than the ATPO, there is no other known bone in that area. Additionally, an ATPO is similar in colour, texture, and composition to bone and, simply put, looks like bone. An ATPO differs from non-calcified/non ossified auricular cartilage as the latter is more flexible, lighter in colour, and exhibits a smooth, glossy surface that resembles old ivory. Histologically, the ATPO shares features of bone and elastic cartilage in that it contains elastic fibres and fits the broad definition of an ossicle as “any small bone” or “a small bone or bone-like structure” [28]. It further fits the definition of an “accessory bone” in that it is a secondary ossification centre that remains separate from the adjacent bone [29]. Grossly, ATPO is a hardened structure of varying size and shape that is loosely or firmly attached to the anterior, posterior, or inferior–lateral surface of the tympanic plate. While all human auricles possess elastic cartilage, not all auricles possess ATPOs that transform from flexible cartilage to brittle bone.
